# High prevalence of DS-1-like rotavirus infection in Thai adults between 2016 and 2019

**DOI:** 10.1371/journal.pone.0235280

**Published:** 2020-06-25

**Authors:** Jira Chansaenroj, Watchaporn Chuchaona, Fajar Budi Lestari, Siripat Pasittungkul, Sirapa Klinfueng, Nasamon Wanlapakorn, Sompong Vongpunsawad, Chintana Chirathaworn, Yong Poovorawan

**Affiliations:** 1 Center of Excellence in Clinical Virology, Faculty of Medicine, Chulalongkorn University, Bangkok, Thailand; 2 Department of Microbiology, Faculty of Medicine, Chulalongkorn University, Bangkok, Thailand; Universidad de la Republica Uruguay- CENUR Litoral Norte Sede Salto, URUGUAY

## Abstract

Rotavirus infection is the most common cause of viral diarrhea in infants and young children but uncommon and usually asymptomatic in adults. In the winter of 2017–2018, a large-scale outbreak of rotavirus in both children and adults was reported in Thailand. The current study focused on the prevalence, genotyping, and molecular characterization of rotavirus infections in Thai adults from July 2016 to December 2019. In 2,598 stool samples collected from adult residents of Bangkok (aged #x2265; 15 years) with acute gastroenteritis, rotavirus was detected via real-time RT-PCR analysis of the *VP6* gene. *G*, *P* and *I* genotypes were determined by direct sequencing of *VP7*, *VP4*, and *VP6* genes, respectively. Our results showed 8.7% (226/2,598) of stool samples were positive for rotavirus. The incidence of rotavirus was high during the winter season of 2017–2018 (17.7%) compared to another studied periods (4.5% between July 2016- October 2017 and 2.8% between March 2018- December 2019). Nucleotide sequencing of *VP7* and *VP4* revealed *G3P[8]* as the predominant strain (33.2%,75/226), followed by *G9P[8]* (17.3%,39/226), and *G2P[4]* (15.0%,34/226). Uncommon G and P combinations were additionally detected at low frequencies. *VP6* sequencing was conducted to discriminate *I* genotype between the Wa and DS-1 genogroup. The unusual DS-1-like *G3P[8]* strain was most prevalent amomg rotavirus strains detected in this study (29.6%, 67/226), and the corresponding *VP7* sequences showed high nucleotide identity with unusual DS-1-like globally circulating strains. Our study demonstrates that rotavirus outbreaks in adults are attributable not only to high prevalence of RV infection but also the unusual DS-like genogroup. The collective findings reinforce the importance of investigating rotavirus diagnosis in adults suffering from acute gastroenteritis and taking appropriate preventive measures.

## Introduction

Rotavirus (RV) is a common pathogen associated with acute viral gastroenteritis in humans worldwide, especially in developing countries [1]. Although significantly more prevalent in young children, RV infection has been reported in parents and caretakers of children [2]. RV can transmit within families and between adults caring for children with RV infection, as evidenced by serological tests [3, 4]. Adults with RV infection may either be asymptomatic or present with mild to severe symptoms [5]. The incidence of rotavirus infections in adults varied between 3–18% with a high prevalence during the winter to spring months (November to March in the northern hemisphere) [6, 7]. Individual may have acquired immunity from previous natural exposures during childhood, leading to a lower incidence of infection in adulthood.

RV is a double-stranded RNA genome virus. RV belongs to the *Reoviridae* family and is classified into 10 species (A-J) based on antigenic and genetic variants of viral protein 6 (*VP6)*. Among the species, Rotavirus group A (RVA) is commonly found in children worldwide [8]. The triple-layered viral particle composed of 11 gene segments encoding six structural proteins (VP1 to VP4, VP6, and VP7) and five or six non-structural proteins (NSP1 to NSP5/NSP6) [9]. Genetic characterization of the *VP6* gene is used to differentiate between RV groups. VP7 and VP4 comprise the outer layer of the virion and thus form the basis of a binary classification system defining G (glycoprotein) and P (protease) genotypes, respectively. In the genotyping system, the genotype constellation is described by the nomenclature *Gx-P[x]-Ix-Rx-Cx-Mx-Ax-Nx-Tx-Ex-Hx*, which defines the genotypes of *VP7-VP4-VP6-VP1-VP2-VP3-NSP1-NSP2-NSP3-NSP4-NSP5*, respectively, with x indicating the number of corresponding genotypes [10]. At present, 36 *G*, 51 *P*, 26 *I*, 22 *R*, 20 *C*, 20 *M*, 31 *A*, 22 *N*, 22 *T*, 27 *E* and 22 *H* genotypes among the RVA have been identified in human and animal species worldwide [11]. To date, *6 G* genotypes (*G1-G4*, *G9*, and *G12*) and *3 P* genotypes (*P[4]*, *P[6]*, and *P[8]*) have been commonly identified within the *G-P* combinations of RVA. In humans, the predominant RV genotypes are *G1P[8]*, *G2P[4]*, *G3P[8]*, *G4P[8]*, *G9P[8]*, and *G12P[8]*, which often vary by region and time and are particularly prevalent in developing countries [8]. In humans, the major RV strains consist of the Wa-like genogroup (*G1*, *G3*, *G4*, *G9*, or *G12-P[8]-I1-R1-C1-M1-A1-N1-T1-E1-H1*), DS-1-like genogroup (*G2-P[4]-I2-R2-C2-M2-A2-N2-T2-E2-H2*) and the less common AU-1-like genogroup (*G3-P[9]-I3-R3-C3-M3-A3-N3-T3-E3-H3*) which have origins in distinct animal species. The segmented nature of the genome enables both intra- and inter-genogroup reassortment between/within animal and human strains, which can lead to the wide spread among humans. Interspecies reassortment derived from zoonotic transmission increase the genetic diversity among circulating RV and the incidence of acute gastroenterisits in human [12]. Recently, the unusual DS-1-like inter-genogroup reassortment strains possessing genotype constellations *G1/3/8/9-P[8]-I2-R2-C2-M2-A2-N2-T2-E2-H2* have emerged and spread among human populations in at least five continents (Asia, Australia, Europe, North America, and South America) [11, 12].

At present, two live oral RV vaccines, Rotarix (GlaxoSmithKline Biologicals S.A., wavre, Belgium; RV1, *G1P[8]*) and RotaTeq (Merck&Co., Inc., Whitehouse, NJ; RV5, *G1-G4*, and *P[8]*), with high efficacy, are licensed in more than 100 countries. However, vaccine efficacy clearly varies among different populations and drives the evolution of more dynamic and diverse wild-type strain populations in the post-vaccine era [13–15]. Moreover, introduction of RV vaccines may enforce additional selective pressure on currently circulating strains, resulting in the generation and spread of novel RV strains worldwide [16]. The above RV vaccines were licensed as optional vaccines in Thailand in 2006 and 2008, respectively [17]. However, RV outbreaks in Thailand still occurred among children after introduction of the vaccines, with different genotypes playing major roles in individual epidemics [18–20].

In Thailand, RV gastroenteritis is common in children but not as prevalent in adults. In the winter season of 2017–2018, an outbreak of acute RV-induced gastroenteritis in adults was reported in Thailand (mainly Bangkok). As mentioned earlier, immunity acquired following exposure during childhood may effectively contribute to protection against infection in individual adults. Therefore, the potential factors underlying the increased number of adults suffering from RV infections is worth investigation. The main objective of the current study was to determine the genotype distribution of RV in adults over an extended period from July 2016 to December 2019.

## Materials and methods

This study was approved by the Institutional Review Board (IRB) of the Faculty of Medicine, Chulalongkorn University (IRB 634/59; Bangkok, Thailand). The director of King Chulalongkorn Memorial Hospital authorized the use of stored samples. All clinical samples were investigated anonymously. The IRB waived the requirement for written informed consent.

### Specimen collection

From July 2016 to December 2019, 4,472 stool specimens from routine testing service for acute gastroenteritis at King Chulalongkorn Memorial Hospital and Bangpakok 9 International Hospital (Bangkok, Thailand) were collected. Of these, 2,598 stool specimens from hospitalized and outpatient adults with acute gastroenteritis (≥15 years) were used for this study (1,058 males and 1,540 females). Enrolled patients presented with acute gastroenteritis, i.e. three or more loose liquid stools per day, along with moderate to severe dehydration with or without fever and vomiting. Available information of patients included sex, age, and collection date.

### Rotavirus detection

Viral RNA was extracted from 10% (v/v) stool suspension in phosphate buffer saline. RNA was automatically extracted from 200 μL supernatant stool samples after centrifugation at 4,000 *x* g for 10 minutes using a magLEAD 12gC instrument (Precision System Science, Chiba, Japan) with a magLEAD Consumable Kit (Precision System Science, Chiba, Japan) according to the manufacturer’s instructions. Amplification of the partial *VP6* gene was performed in a 25 μL reaction volume comprised 10 μM forward primer VP6-F (5’- GACGGVGCRACTACATGGT-3’) and reverse primer VP6-R (5’-GTCCAATTCATNCCTGGTGG -3’), based on the study of Kang G. et al. [21] and 2 μL RNA template using the QuantiTect SYBR Green 1-step real-time RT-qPCR Kit (Qiagen, Hilden, Germany). Reverse transcription was conducted at 50°C for 30 min, followed by heat denaturation at 95°C for 15 min. In total, 45 cycles of amplification were performed comprising denaturation at 94°C for 15 sec, annealing at 50°C for 30 sec, and extension at 72°C for 30 sec. Melting curve analysis was performed from 60°C to 95°C with 1°C increments. Amplified products were purified and sequenced for I genotype identification.

### Co-infections of diarrhea-inducing viruses

To identify cases of co-infection, RV RNA was tested along with those of other gastroenteritis viruses, such as norovirus, astrovirus, sapovirus, bocavirus, enterovirus, adenovirus and parechovirus. Detection of other gastroenteritis viruses was performed by polymerase chain reation (PCR) or reverse-transcription PCR (RT-PCR) as previously described (norovirus [22], astrovirus, sapovirus, enterovirus [23], parechovirus, bocavirus [24] and adenovirus [25]).

### Sequence determination of *G*, *P*, and *I* genotype of rotavirus and phylogenetic analysis

Samples initially tested positive based on *VP6* screening were further subjected to amplification of genes encoding the structural proteins VP6, VP7, and VP4 using the SensiFAST 1-step RT-PCR kit (Bioline, London, UK). Consensus primer pairs specific for the *VP6* gene from the study of Theamboonlers *et al*. [26] were used for amplification. The consensus primer pairs of BEG9/END9 and con2/con3 were used for amplification of *VP7* [27] and *VP4* [28], respectively. The 25 μL RT-PCR mixture contained 2X buffer, 10μM of consensus forward and reverse primers and 2 μL RNA. Reverse transcription steps included incubation at 45°C for 30 min, followed by 94°C for 5 min. PCR conditions were as follows: 40 cycles of denaturation at 94°C for 30 sec, annealing at 55°C for 45 sec, and extension at 72°C for 2 min. Amplicons were agarose gel-purified and sequenced by FirstBASE Laboratories (SDN BHD, Salangor, Malaysia).

Sequencing data were analyzed using Chromas 2.23 (Technelysium, Queensland, Australia). Nucleotide sequences identity was analyzed via BLAST. *G (VP7)*, *P (VP4)*, and *I (VP6)* genotypes were validated using the online RotaC rotavirus genotyping tool (RotaC version 2) [29].

Nucleotide sequences were aligned using Clustal Omega (www.ebi.ac.uk/Tools). Phylogenetic trees were constructed and molecular evolutionary analyses were conducted using MEGA 6.0 software. The Kimura-2 correction parameter model and Maximum likelihood method were applied to construct the phylograms of *VP7*, *VP4*, and *VP6*. The best-fit models were determined through testing the model parameter. Tree robustness was determined by bootstrapping (1,000 replicates), with bootstrap values >70% considered significant.

Nucleotide sequences were deposited in the GenBank database under the accession numbers MN836856-MN837068 and MN989603-MN989613 for *VP7*, MN989625-MN989850 for *VP6*, and MN837284-MN837475 and MN989614-MN989624 for *VP4*.

### Statistical analyses

Statistical analyses were performed using IBM SPSS statistic for Windows, version 21 (IBM Corp., Armonk, NY). The analysis was conducted by dividing individuals into three age groups (15–29, 30–59 and > 59years old). Rate of RV infection was compared between three periods of time (July 2016-June 2017, July 2017-June 2018, July 2018-December 2019). A Chi-square table was used to compare the percentages of infected individuals between age groups and studied periods. All comparisons were two-sided and *p*-value < 0.05 was considered to be statistically significant.

## Results

**Fig 1:**
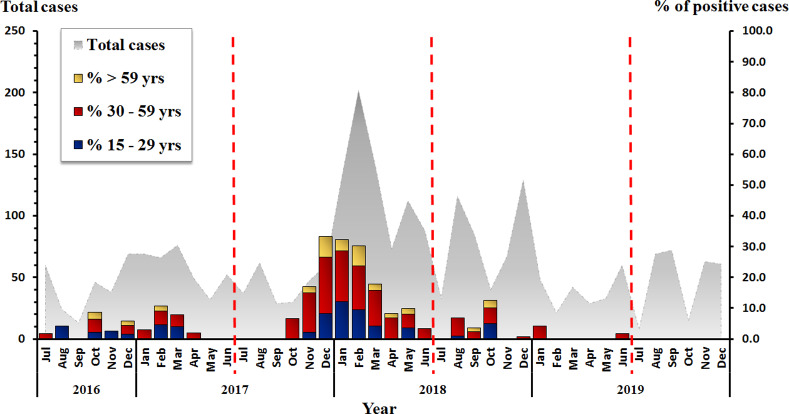
Percentages of rotavirus infection classified by age group between July 2016 and December 2019. The monthly number of samples from adults with acute gastroenteritis is shown in gray (left scale). Bar graphs show the percentage of RV-positive cases (right scale).

In this study, specimens from patients aged between 15–96 years old were investigated. The mean and median age of patients were 46.7 and 44.0 years, respectively. Among the 2,598 stool samples from adults (≥15 years) with acute gastroenteritis, 8.7% (226/2,598) were positive for RV based on the real-time RT-PCR *VP6* primary screening assay (74 male and 152 female). The prevalence of RV infection was high during the winter season (November–March) as shown in Fig 1. Between July 2017 and June 2018, the prevalence of rotavirus infection in adults was highest among the three-year study period and accounted for 17.7% (179/1013). The rate of RV infection was highest in the 30–59 year age group (56.6%, 128/226), followed by 15–29 year age group (28.3%, 64/226), and ≥ 59 year age group (15.1%, 34/226) as shown in Fig 1 (*p*-value < 0.001).

Among the total RV-positive samples, 85.0% (192/226) were solely infected with RV while 15.0% (34/226) of cases showed mixed infection with other diarrhea-inducing viruses. The major co-infecting virus was norovirus which accounted for 7.1% (16/226) followed by astrovirus 2.7% (6/226), bocavirus 2.7% (6/226), sapovirus 0.9% (2/226), and enterovirus 0.9% (2/226). Co-infection with adenovirus was not found. Moreover, triple infection with RV, astrovirus and bocavirus was detected in one patient. Bacterial pathogens and other causative agents not mentioned above were not investigated.

**Fig 2:**
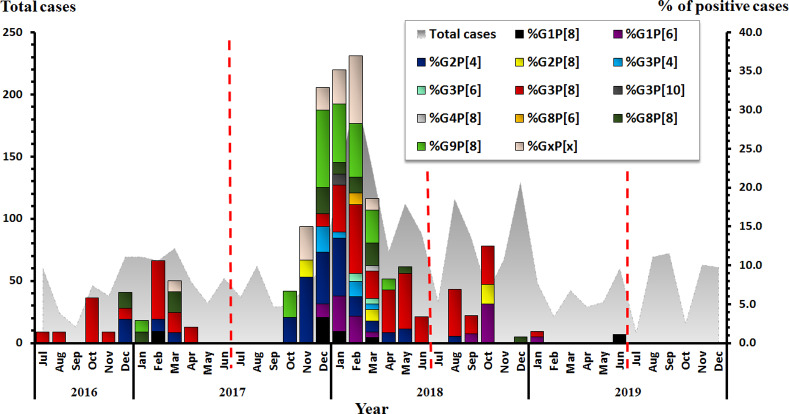
Distribution of rotavirus *G* and *P* genotypes between July 2016 and December 2019. The monthly number of samples from adults with acute gastroenteritis is shown in gray (left scale). Bar graphs show the percentage of RV-positive cases (right scale).

Based on the sequence of *VP6*, an unusual DS-1-like backbone (*G1/3/8/9-P[4/6/8]-I2*) was detected predominately in 48.2% (109/226) of RV-positive samples, followed by Wa-like strain (*G1/3/8/9-P[6/8]-I1*) (31.9%, 72/226), usual DS-1-like strain (*G2-P[4]-I2*) (19.4%, 44/226), and AU-1-like strain (*G3-P[10]-I3*) (0.4%, 1/226). When analyzed together with the distribution of *G* and *P* types, our results showed that *G3P[8]* (33.2%, 75/226) (unusual DS-1-like *G3P[8]* (29.6%, 67/226) and Wa-like *G3P[8]* (3.5%, 8/226)) was the most common genotype, followed by *G9P[8]* (17.3%, 39/226) (Wa-like *G9P[8]* (16.4%, 37/226) and unusual DS-1-like *G9P[8]* (0.9%, 2/226)), *G2P[4]* (15.0%, 34/226), and *G1P[6]* (8.4%, 19/226). Uncommon G and P types were additionally detected at low frequencies such as *G3P[4]* (3.5%, 8/226), *G3P[10]* (0.4%, 1/226), and *G4P[8]* (0.4%, 1/226) (Fig 2).

**Fig 3:**
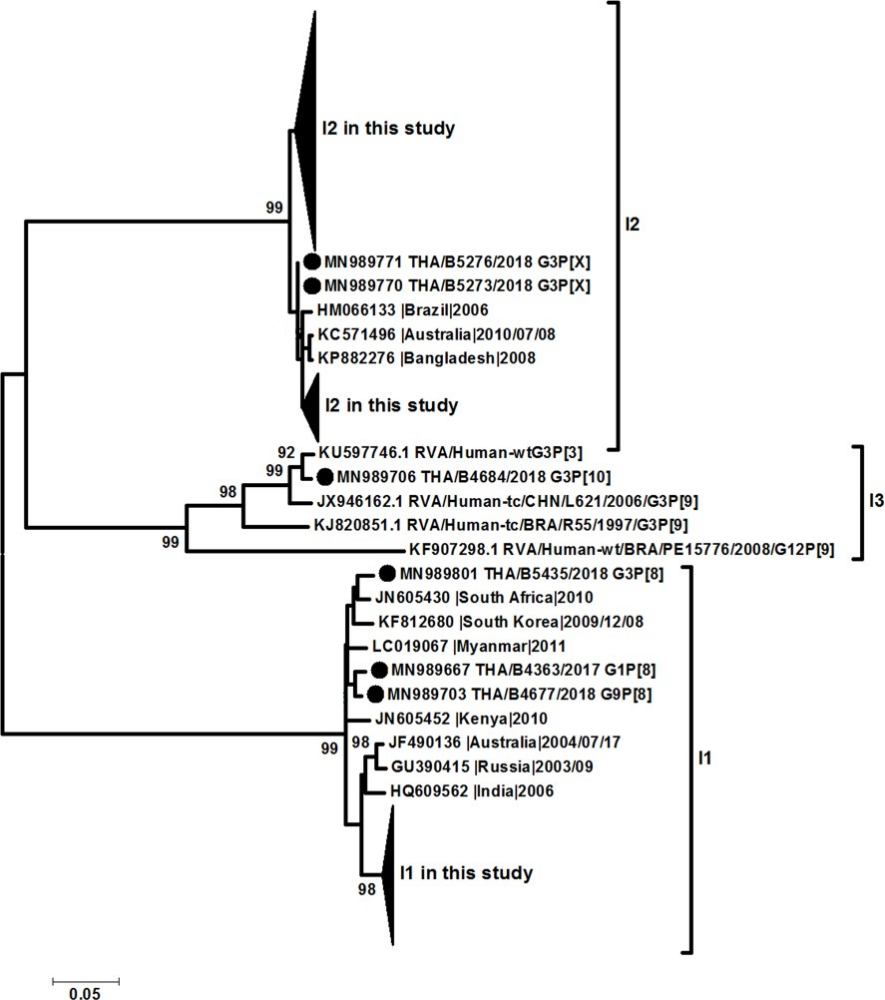
Phylogenetic tree of the *VP6* gene. Evolutionary history was inferred using the Maximum Likelihood method based on the Kimura 2-parameter model. The tree is drawn to scale, with branch lengths measured by the number of substitutions per site. The analysis involved 252 nucleotide sequences. All positions with <95% site coverage were eliminated.

**Fig 4:**
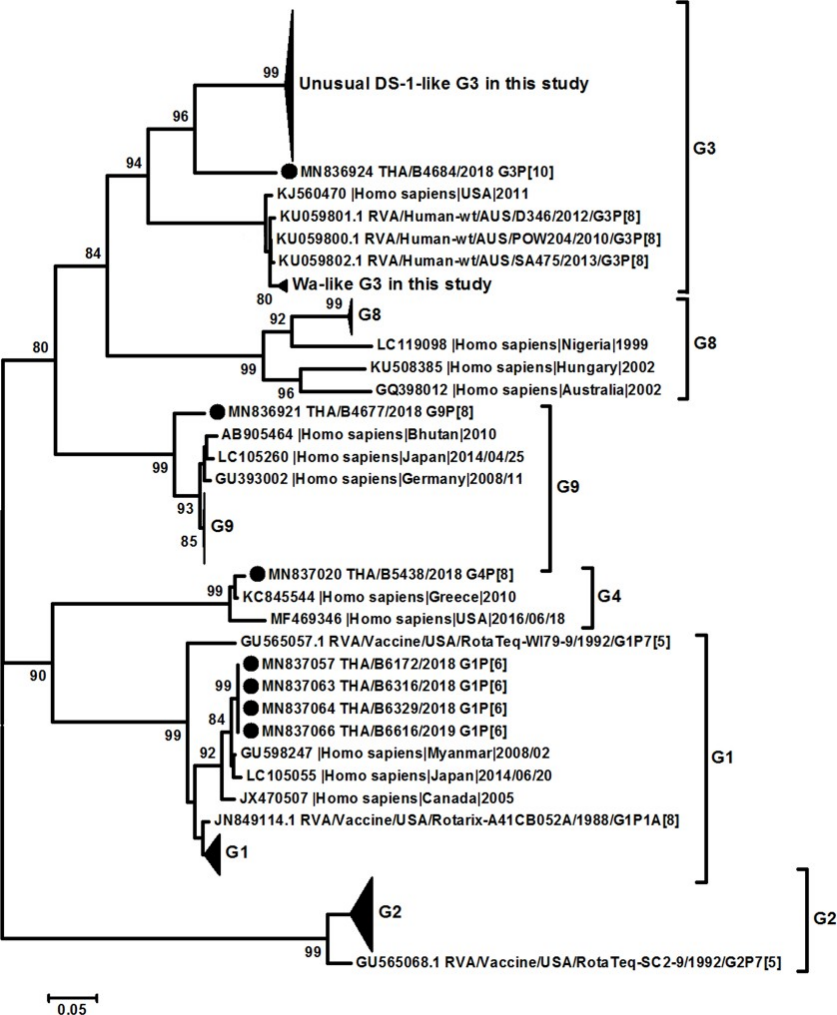
Phylogenetic tree of the *VP7* gene. Evolutionary history was inferred using the Maximum Likelihood method based on the Kimura 2-parameter model. The tree is drawn to scale, with branch lengths measured by the number of substitutions per site. The analysis involved 252 nucleotide sequences. All positions with <95% site coverage were eliminated.

**Fig 5:**
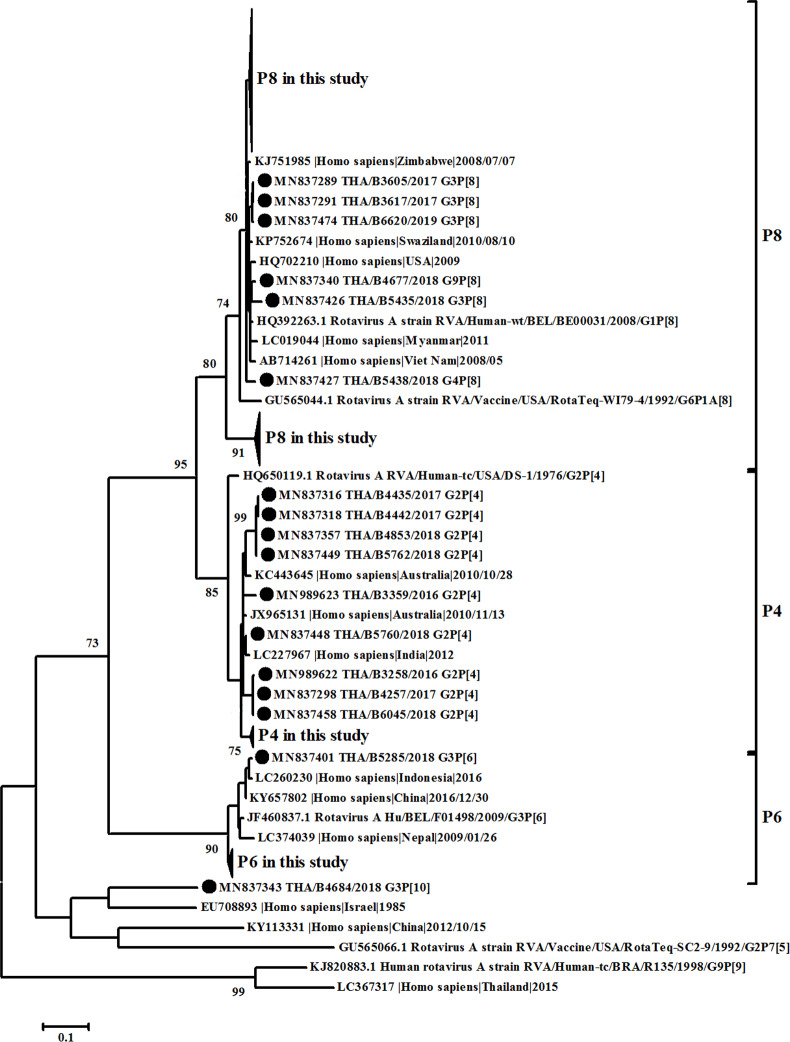
Phylogenetic tree of the *VP4* gene. Evolutionary history was inferred using the Maximum Likelihood method based on the Kimura 2-parameter model. The tree is drawn to scale, with branch lengths measured by the number of substitutions per site. The analysis involved 185 nucleotide sequences. All positions with <95% site coverage were eliminated.

All samples positive for rotavirus *VP6* screening can be sequenced for complete *VP6* genome, whereas 99.1% (224/226) and 89% (201/226) of samples can be sequenced for complete *VP7* and *VP4* gene, respectively. The phylogenetic analysis of *VP6* (I type) led to identification that sequences obtained in this study were clustered in 3 groups, Wa-like (I1) (31.9%, 72/226), DS-1-like (I2) (67.7%, 153/226), and AU-1-like (I3) (0.4%, 1/226) (Fig 3). On the other hand, the phylogenetic analysis of *VP7* gene showed the different clustering of between Wa-like *G3* and unusual DS-1-like *G3* which clustered in different lineages (Fig 4). The percentage of nucleotide identity between Wa-like and unusual DS-1-like *G3* was 81% while amino acid identity was 91%. Multiple amino acid variations were detected, especially in antigenic regions C (T212A and N213T) and F (N238D and N242A). The *P[8] VP4* gene did not cluster in the different lineages and correlate with the phylogenetic tree of *VP7* (Fig 5).

## Discussion

In this study, the molecular characteristics of RV in adult patients with gastroenteritis in Bangkok, Thailand were investigated over a 3-year period (July 2016– December 2019) using a large number of stool specimens collected from various age groups. The RV genotype *G3P[8]I2* (unusual DS-1-like *G3P[8]*) was the most dominant strain (29.6%), followed by *G9P[8]I1* (Wa-like *G9P[8]*; 16.4%) and *G2P[4]I2* (DS-1-like *G2P[4]*; 15.0%). The previous report from six provinces in Thailand showed that *G3P[8]I2* has emerged in Thailand since 2015 [20]. The positive rate of rotavirus infection in adults presenting with acute gastroenteritis in this study was 8.7%. This rate was similar to some of the previous reports in Thailand [20, 30], but lower than a study in Bangkok which had a relatively small sample size [19]. Nevertheless, all of the previous studies agreed that the incidence of RV infection in adults increased during the winter season (November–March) indicating a similar seasonality pattern to that reported in children population [18, 31, 32]. Apart from being seasonal-specific, rotavirus gastroenteritis can be transmitted from children to adults [2–4]. Unfortunately, our study did not include the history of contact with sick children, therefore, the burden of RV gastroenteritis from children-to-adult transmission cannot be determined. The results of this study showed that rotavirus was an important agent for acute gastroenteritis in adults, and should not be underestimated.

In addition to the prevalence of adult RV infections, the molecular characteristics of RV-induced acute gastroenteritis in adults were investigated. The distribution of *G* and *P* types in Thailand have changed over time, but seasonality of RV infection remains unchanged [33]. The *G1* genotype combination with *P[8]* strains has been predominantly identified in many parts of Thailand since 1993, followed by genotypes *G2*, *G3*, and *G9* in different seasons and regions [34]. From 2000 to 2016 [20, 35], various *G* and *P* type combinations have been detected, such as *G1P[8]*, *G2P[4]*, *G3P[8]*, and *G9P[8]*. On an annual basis, the dominant *G* and *P* types were identified as *G9P[8]* from 2000 to 2004, *G1P[8]* from 2005 to 2009, *G3P[8]* from 2009 to 2011, *G1P[8]* from 2012 to 2014, and *G9P[8]* from 2015 to 2016. Moreover, uncommon genotypes, such as *G3P[10]* and *G12P[8]*, were detected. In the current study, *G3P[8]* was the predominant strain (33.2%, 75/226), consistent with previous reports from Indonesia (2015–2016) [36] and Brazil (2016–2017) [37, 38], followed by *G9P[8]* (17.3%, 39/226) and *G2P[4]* (15.0%, 34/226). In terms of genetic backbone, the DS-1-like strain (I2) was prevalent (55.3%, 125/226) with the highest being unusual DS-1-like *G3P[8]I2* (53.6%, 67/125), followed by *G2P[4]*I2 (27.2%, 34/125).

The effects of vaccination on distribution patterns of co-circulating RV strains have been investigated. A previous study in Australia reported that the introduction of RV vaccines affected the ecology of RV by increasing diversity and differences in genotype predominance. Increased prevalence of *G12P[8]* across states was observed following the use of RotaTeq and *G2P[4]* and DS-1-like *G3P[8]* with the use of Rotarix [39]. Another study in Japan showed that the dominant genotype shifted from *G3P[8]* to *G1P[8]* and *G2P[4]* after vaccine introduction [40]. In the previous report in Thailand [20, 41], RV with DS-1-like backbone (*G1*,*3*,*4*,*9P[8]I2*) has been prevalent in children and adults since 2013, with a trend of continued significant increase until 2016 in distinct provinces. In the present study, unusual DS-1-like *G3P[8]* was identified as the major strain in Thailand. The sequences of *VP7* and *VP4* genes displayed high percentage nucleotide identity to the Australian unusual DS-1-like *G3P[8]* strains circulating between 2006 and 2011 [12]. This unusual DS-1-like *G3P[8]* strain has been identified in several countries with a range of vaccine coverage, with different rates of infection depending on geographic and temporal patterns, including Australia, Germany, Hungary, USA, Brazil, Japan, and Thailand [10–12, 20, 36, 38, 42–46], indicating that the DS-1-like backbone is derived from a globally circulating pool of RV. Studies on children infected with the unusual DS-1-like *G1P[8]* strain revealed no significant differences in clinical severity relative to disease caused by Wa-like *G1P[8]* [47, 48]. However, these strains contain several different amino acids, especially in the antigenic region, which may affect vaccine efficacy, as shown previously for unusual DS-1-like *G1P[8]* [49]. This reassortment has a considerable impact on communities, potentially conferring some advantage to viral propagation and survival, although severity of diarrhea may not be affected. While no data on effectiveness of vaccines against unusual DS-1-like *G3P[8]* are available at present, the efficacy of the RV vaccine against Wa-like *G1P[8]*, unusual DS-1-like *G1P[8]*, *G2P[4]* and Wa-like *G9P[8]* strains has been confirmed based on mean Vesikari score and distribution of Vesikari score [48–52].

The main limitation of this study is the lack of clinical severity and RV vaccination history of patients and their families. Although rotavirus vaccines were licensed in Thailand as optional vaccines since 2006, the vaccine coverage was low. In January 2020, Thailand implemented rotavirus vaccine to all infants. Vaccine introduction was not likely to be the factor contributing to the prevalence of the unusual DS-1-like genogroup in this study and the underlying reason remains to be established. Although our study subjects were mainly Bangkok resident which may not represent the overall national population, data were obtained from a large number of patients.

In conclusion, the increase in RV infections in adults during the winter season in 2017–2018 relative to that during the equivalent periods in the previous and subsequent years was of significant interest. Genotyping of the RV backbone in routine surveillance provided additional information to explain this finding. The unusual DS-1-like *G3P[8]* strain was most prevalent amomg rotavirus strains detected in this study (29.6%, 67/226). The results obtained may aid in further understanding of not only viral evolution but also symptomatic infection in adults. The effectiveness of the vaccine should be concerned for the unusual DS-1-like strain. Therefore, continuous and extensive surveillance, including genotyping of RV infection, is essential for preventive measures, vaccine efficacy, and control of viral gastroenteritis outbreaks.
